# Clinical research nurse predictions of trial failure, recruitment and retention: a case for their early inclusion in trial design

**DOI:** 10.1186/s13063-023-07504-9

**Published:** 2023-07-18

**Authors:** Frances Shiely, Danielle Murphy, Seán R. Millar

**Affiliations:** 1 Trials Research and Methodologies Unit, HRB Clinical Research Facility, University College Cork, Cork, Ireland; 2 HRB Trials Methodology Research Network (HRB TMRN), Cork, Ireland; 3 School of Public Health, University College Cork, Cork, Ireland

**Keywords:** Recruitment, Retention, Clinical research nurse, Trial methodology

## Abstract

**Background:**

Clinical research nurses are a key part of the clinical trial team but typically get involved later in the trial, usually during recruitment. The purpose of our study was to establish if CRNs who read the trial protocol can predict the performance of the trial.

**Methods:**

We randomly selected 18 trial protocols with three statuses, terminated, withdrawn, and completed, from ClinicalTrials.gov, between 2014 and 2018 inclusive. We gave the protocols to five CRNs, asked them to make a judgement and provide a reason for that judgement (via a 12-item questionnaire) on the status of the trial (terminated, withdrawn or completed), if the trial met its recruitment target, if it recruited on time, and if it retained its participants. We also asked if it was likely a CRN was involved in the design of the trial. The CRNs were blinded to the study outcomes, did not receive any training on how to read a protocol and were prohibited from using/abstained from using the internet while completing the task.

**Results:**

Twenty-three questionnaires on 23 trial protocols (18 different trials) were completed by 5 CRNs. The CRNs correctly predicted the trial status 48%, 95% CI: 29–67% (11/23) of the time; successful/unsuccessful recruitment 74%, 95% CI: 54–87% (17/23) of the time; on-time recruitment 70%, 95% CI: 49–84% (16/23) of the time; and participant retention 52%, 95% CI: 33–71% (12/23). CRNs identified 100% (sensitivity) of sites that hit their target and 63%, 95% CI: 36–84% (specificity) of sites that missed their target.

**Conclusions:**

CRNs are very good judges of trial recruitment and site performance issues and are a vital part of the clinical trial team. Taken with the ESP (Estimating Site Performance) study, we have made a strong case for broadening the trial team at the trial design stage. Early engagement of a broad skillset can potentially offset problems of recruitment, retention and trial failure.

**Supplementary Information:**

The online version contains supplementary material available at 10.1186/s13063-023-07504-9.

## Background

Clinical research nurses (CRNs) are pivotal members of the clinical trial team [1]. They are usually associated with trial recruitment, data collection, assisting in the day-to-day management of the trial and caring for the research participants [2, 3]. The role of the CRN varies from country to country and so too does their title — clinical research nurse, clinical trial nurse, research coordinator or clinical trial coordinator. In Ireland, for example, the CRNs fulfil the trial manager role also, as there is no recognised trial manager role within the Health Service Executive (Irelands National Health Provider). The UK Clinical Research Collaborative’s Subcommittee for Nurses in Clinical Research defined a CRN as ‘a nurse who is employed principally to undertake research within the clinical environment’ and specifically includes nurses working under the direction of health researchers, those supporting research in a major facility, and those undertaking their own research [4]. CRNs are ‘qualified nurses’, most commonly with BSc General Nursing but also, General and Children’s, Intellectual Disability, Mental Health, Midwifery. There are no undergraduate degree programmes for clinical research nurses in Ireland or the UK. Some CRNs, though it is not mandatory, will pursue postgraduate courses in clinical research or clinical trials, though there is none specifically designed for CRNs.

Successful recruitment and retention of participants to clinical trials are identified as two of the most difficult aspects of the trial process [5–9]. Approximately 80% of clinical trials fail to recruit to their initial timeline and targets, a figure which highlights the significant challenge for trial teams globally [10, 11]. When trials fail to recruit within the envisioned timescale, it leads to an increase in cost, or they fail to reach their required sample size giving the potential for underpowered studies [5, 6, 12–14]. Retention is equally important, as participant drop-out or incomplete data can cause problems in the analysis, interpretation, and external validity of the results [6, 15]. There is little available evidence for effective retention strategies [7] and this is costing trial teams, and by extension the funders, thousands of Euro/Pounds [16].

CRNs can potentially influence the trial processes but the scope and contribution of their role to clinical trials is not known [2]. Furthermore, their first encounter with the trial is often late in the overall project, and they rarely have input into the clinical trial protocol [17]. Prior research has shown that trial managers are quite successful at predicting if trial sites would recruit to target [18]. Ten trial managers made predictions for 56 site visits recruiting to eight trials. Trial managers’ sensitivity was 82% and their specificity was 32%, correctly identifying 65% of sites that would hit their recruitment target and 54% of those that did not [18]. We believe CRNs are similarly well placed to provide trial teams with information on potential recruitment and retention to trials and the suitability of the recruitment and retention strategies within the trial protocol. The purpose of our study was to establish if CRNs who read a trial protocol can predict the performance of the trial. We focused on four areas key to the integrity of a trial: the status of the trial (terminated, withdrawn or completed), if the trial met its recruitment target, if it recruited on time, if it retained its participants. We also asked if it was likely a CRN was involved in the design of the trial.

## Methods

### Trial protocol selection

FS searched clinicaltrials.gov in June 2020 for trials with both protocol and results published between 2014 and 2018. We sought studies from each of the three clinicaltrials.gov trial status categories, i.e. terminated, withdrawn and completed. We set ourselves a target sample size of 18 protocols for inclusion because we wanted a sample that was large enough to say something meaningful but not so large that CRNs would be overwhelmed by the number of protocols they were asked to review (Table 2), particularly given that some protocols were very long (Shortest: 6 pages; Longest: 128 pages). Eighteen seemed like a reasonable compromise between sample size and feasibility. We excluded the following: studies of dosing validations; studies that had an agreement with the PI and the sponsor that restricts the PI’s right to discuss or publish trial results after the trial is completed; safety trials; non-randomised trials; single group assignment; studies withdrawn due to lack of funding; no reason for the termination or withdrawal provided. DM randomly selected trials from each of the terminated, withdrawn and completed categories, and assessed them for inclusion. If a trial was found to not be eligible, the next trial on the randomised list was examined for inclusion. This was repeated until our target of 18 trials was reached (Table 1).

**Table 1:** Search strategy

Trial status	Trials identified	Trials randomised for use in the study
Terminated studies	167	9
Completed studies	1066	5
Withdrawn	17	4

We had anticipated 6 trial protocols in each of the terminated, withdrawn and completed categories, however, only four suitable withdrawn studies were available from the 17 identified within the 5-year time period. We thus included more terminated trials than originally anticipated to make up the deficit so our target sample size of 18 was met (Table 1). Our final list of trial protocols is included in Table 2 including the clinicaltrials.gov registration number, title of the trial and the reason for the termination or withdrawal.

**Table 2:** Included trial protocols

Trial ID	Title	Status	Reason
NCT03723577	An Evaluation of a Fibrillar Collagen Dressing to Treat Chronic, Stalled Lower-extremity Wounds	Withdrawn	No subjects enrolled at site
NCT03523299	Pilot Study to Define the Immune Response Following Cryoablation of Invasive Breast Cancer	Withdrawn	Principal investigator wishes to revisit design and start a new study)
NCT03357042	Evaluation of a Physical Intervention for Persistent Post-Concussive Symptoms	Withdrawn	researchers did not find participants to meet the inclusion and exclusion criteria for the testing protocols
NCT03323710	Study of Propranolol Plus Sunitinib in First-line Treatment of Metastatic Renal Cell Carcinoma	Withdrawn	Poor patient recruitment
NCT03069677	Music vs Midazolam During Preop Nerve Block Placement	Completed	
NCT02979899	Trial of TRC105 and Pazopanib Versus Pazopanib Alone in Patients With Advanced Angiosarcoma (TAPPAS)	Completed	
NCT02942576	Edoxaban Treatment Versus Vitamin K Antagonist (VKA) in Patients With Atrial Fibrillation (AF) Undergoing Catheter Ablation (ELIMINATE-AF)	Completed	
NCT02866175	Edoxaban Treatment Versus Vitamin K Antagonist in Patients With Atrial Fibrillation Undergoing Percutaneous Coronary Intervention (ENTRUST-AF-PCI)	Completed	
NCT02861534	A Study of Vericiguat in Participants With Heart Failure With Reduced Ejection Fraction (HFrEF) (MK-1242–001) (VICTORIA)	Completed	
NCT02652156	TAP Block for Postoperative Pain Control	Terminated	Operational barriers at the site
NCT02460991	A Study of ONCO-DOX in Locally Advanced Hepatocellular Carcinoma (SOLACE)	Terminated	Slow enrolment
NCT02411084	Study of BEGEDINA® vs "Conventional Treatment" for Treating Steroid-Resistant Acute GvHD	Terminated	Insufficient rate of accrual
NCT02422446	Effects of Eicosapentaenoic Acid on Endothelial Function in Diabetic Subjects	Terminated	Difficulty enrolling patients with elevated triglycerides under statin treatment
NCT02904265	Efficacy Study of Acetazolamide Versus Diazepam in Continuous Spike and Wave/Landau-Kleffner Syndrome	Terminated	Lack of enrolment
NCT02111785	Dexamethasone Versus Burr Hole Craniostomy for Symptomatic Chronic Subdural Hematoma (DECS)	Terminated	Accrual too slow; study P.I. passed away
NCT01598896	Combination of Dronabinol and Clonidine for Cannabis Dependence in Patients With Schizophrenia (DCCS)	Terminated	Low enrolment due to limited resources
NCT03716050	The Effect of Nitroglycerin Ointment, Fluorescent Angiography, and Incisional Negative Pressure Wound Therapy on Mastectomy Skin Flap Perfusion-Related Problems	Terminated	PI decision due to slow accrual
NCT02044510	Urodynamic and Clinical Efficacy of Mirabegron for Neurogenic Bladder Patients	Terminated	Slow recruitment and small observed effect size

### CRN recruitment

We invited five CRNs at the HRB Clinical Research Facility at University College Cork to participate in the study, with the expectation that at least four would accept. All five accepted the invitation and we decided to continue the work with all five as this would bring an additional perspective to our work. Three CRNs had 15–20 years’ experience, and two had 7–10 years’ experience, working in clinical research. The HRB Clinical Research Facility serves the South South-West Hospital Group in Ireland, a catchment area of 1.2 million people. We prepared four CRN packs, Packs A, B, C and D. Packs A and B each included 5 trials and Packs C and D had four trials each. Each pack included at last one of each trial status, terminated, withdrawn or completed. As all five CRNs agreed to participate in the study, two CRNs independently completed Pack A. They were asked to read each trial protocol provided and then complete a 12-item questionnaire on the protocol (see Supplementary file 1). We did not train the CRNs on how to read a protocol or provide guidance to them on how to respond to the questions. The CRNs were blinded to the purpose of the study and trial statuses and were asked to read the protocol and make a judgement on the following: status of the trial—terminated, withdrawn or completed; if the trial successfully recruited; if it met its target recruitment; if it recruited on time; if it retained its participants; if they thought a CRN was involved in the design of the trial. The CRNs were asked not to use the internet at any time while completing the questionnaire. Each nurse signed a consent form to participate in the study and a signed statement that they did not use the internet while completing the task.

### Outcomes

Our primary outcomes were the proportion of correct CRN predictions of trials completed, withdrawn, or terminated; trials successfully recruited; trials recruited on time; participants retained. The secondary outcome was the correct predication of CRN involvement in the trial design.

### Statistical methods

Quantitatively we calculated the proportions and the sensitivity and specificity for the predictions. For the positive predictions we calculated the positive predictive value (PPV) and for the negative predictions we calculated the negative predictive value (NPV). Qualitatively we recorded the reasons for their predictions and themed these reasons for each of the five categories.

## Results

### Trial status predictions

Twenty-three questionnaires on 23 trial protocols (18 different trials) were completed by five CRNs. Trials had three statuses: completed (*n* = 7), terminated (*n* = 11), and withdrawn (*n* = 5). We combined the terminated and withdrawn trial predictions when reporting the analysis (*n* = 16); however, when considering a correct prediction, a terminated trial was only correct if it was predicted as terminated, and the same was true for withdrawn. The CRNs correctly predicted the trial status 48%, 95% CI: 29–67%, (11/23) of the time. Of the 7 completed studies, 71% (5/7) were correct (sensitivity) and of the terminated/withdrawn studies, 38%, 95% CI: 16–64%, (6/16) were correct (specificity). In addition to making a judgement on the trial status, CRNs were asked to provide a reason for the judgement. The reasons are themed and direct quotations related to each theme are presented in Table 3.

**Table 3:** CRN reasons for judgements made on trial status

**Themes for completed trials correctly predicted**	**Quotes for CRN’s reasons for judgement**
Simplicity of protocol	- Straight forward protocol & short duration - Not too cumbersome for patients - No additional procedures - Prevalent disease
Planning	- Clear plan for recruitment - Adequate recruitment given the timeframe
Safety	- Drug already on market so assured safety
**Themes for withdrawn/terminated trials correctly predicted**	**CRN reasons for judgement**
Recruitment issues	- Hospital involved not a specialist centre for the specific disease needed for recruitment
Burdensome protocol	- Protocol design seems cumbersome for patients - Protocol poorly designed - Major issues with bias - No clear aim
Restrictive inclusion criteria	- Recruitment problems due to exclusion criteria being too restrictive
Safety	- No mention of ethical approval, - Pharmaceutical evidence lacking

### Successful, and unsuccessful, recruitment predictions

The CRNs predicted if the trial would successfully recruit. Of the 23 predictions made, CRNs were correct 74%, 95% CI: 54–87%, (17/23) of the time. Just over half of predictions were yes (13/23; 57%). Of these, 7 (PPV = 54%, 95% CI: 26–80%) were correct. CRNs identified 100% (sensitivity) of sites that hit their target and 63%, 95% CI: 36–84% (specificity) of sites that missed their target. 10/23 predictions were ‘did not meet target’ and all were correct (NPV = 100%). Table 4 presents the themes and reasons for the CRNs’ judgements on successful, or unsuccessful, trial recruitment.

**Table 4:** CRN reasons for judgements made on successful, or unsuccessful, trial recruitment

**Themes for successful recruitment**	**Quotes of CRN’s reasons for judgement**
Safety	- Drug previously approved so safety assured - Close monitoring - Unrestrictive recruitment, e.g., large disease population - Minimal inclusion criteria - Good timeframe for recruitment - International sites
Patient burden	*-* Study schedule not too busy - Study procedures follow regular check-ups - Patients with disease should have time to come to check-ups due to age
Other	- Minimal other treatment options for disease - Not an IMP study
**Themes for unsuccessful recruitment**	**CRN reasons for judgement**
Restrictive inclusion and exclusion criteria	- Too restrictive
Patient burden	- Too time-consuming for some patients with regular check-ups - Study diary time consuming - Very time-consuming
No. of sites	- Recruitment may be difficult with only one site - Only one site is very restrictive considering the recruitment number
Limited treatment options	- Patients may not choose this treatment option due to vulnerability - Shot in the dark treatment - Little variation in medication - Patients may opt for single-process treatment

### On-time recruitment, or not

Twenty-three predictions were made regarding the trial recruiting on time or as per the proposed schedule. The CRNs were correct 70%, 95% CI: 49–84% (16/23) of the time. Of the 8 positive predictions, 3 were correct (PPV = 38%, 95%CI:10%-74%). Of the 15 negative predictions, 13 were correct (NPV = 87%, 95% CI: 58–98%). Of the sites that were recruited on time, CRNs identified 60%, 95% CI: 17–93% of them (sensitivity). Of those that didn’t recruit on time, CRNs were correct in 72%, 95% CI: 46–89% of cases (specificity). The reasons for predicting a trial would recruit on time or not on time were themed and are presented in Table 5.

**Table 5:** CRN reasons for judgements made on trials recruiting, or not recruiting, on time

**Themes for recruiting on time**	**Quotes for CRN’s reasons for judgement**
Duration	- Study duration too long
Number of sites	- Many sites in varied location
Treatment	- Many patients with minimal treatments options
**Theme for not recruiting on time**	
Over-ambitious recruitment target	- 200 is too ambitious to recruit within a year - Numbers for recruitment too high
Recruitment strategy not appropriate	- Recruitment can only come from elective lists therefore limiting number of participants
Protocol complications	- Unclear protocol - Protocol too complicated - Inclusion and exclusion criteria too strict
Timing	- Not much time for screening potential patients - Tight timelines - Patients do not have enough time to make decisions for serious conditions - Timeline to recruit too short - Start-up delay
Medical complications	- Many patients will not opt to take unnecessary medication - Medication complications - Changing dose of concomitant meds - 6 months long time to stick to these con. med regimes
Ethics	- Ethics delay

### Retention predictions

In terms of participant retention, CRNs made correct predictions 12/23 times (52%, 95% CI: 33–71% of the time). Fifteen predictions were ‘yes’ and 8/15 (PPV = 53%, 95% CI: 27–78%) predicted correctly. Eight predictions were ‘no’ the studies would not retain its recruited patients, 4/8 (NPV = 50%, 95% CI: 17–83%) were correct. The reasons are themed in Table 6 along with examples of the reasons provided.

**Table 6:** CRN reasons for participant retention judgements

**Themes for participants retained**	**CRN reasons for judgement**
Patient burden	- Patients generally come back for specialist care - Only one visit for the procedure - Trial duration short
Withdrawal criteria	- Criteria for withdrawal not extensive
Timeline duration	- Short timeline
**Themes for participants not retained**	
Patient burden	- Time-consuming for patient - Patients are locked into long-term restrictions around concomitant meds - If patient sees no benefit early or worsening, they might self-withdraw
Medication complications	- Changing dose of con. meds
Protocol issues	- Issues with adherence due to specific population
Other	- Nominating patients is too much of a burden on site staff-may deviate from protocol

### CRNs involved in trial design

CRNs were asked if they thought a CRN was involved in the trial design. Of the 23 predictions made, 12/23 (52%, 95% CI: 33–71%) were correct. Most trials did not involve the CRN in the trial design (17/23; 74%). The reasons provided by the CRNs are themed and presented in Table 7.

**Table 7:** Reasons for predicting CRNs were involved in the trial design

Themes for CRN involvement/non-involvement in trial design	CRN reasons for judgement
Protocol specifics	- Detailed protocol in relation to medications and the specifics of adverse events/withdrawal criteria
Patient burden	- It does not appear that allowances have been made for the difficult nature of this trial and how it may affect participants - A CRN would have foreseen the realities of life for these patients - A CRN would foresee issues with patient experience of the catheter
Recruitment issues	- The target recruitment number seems too large to be realistic
Protocol issues	- Poorly designed protocol - No timeframe seen from time of enrolment to last subject completed - The protocol is very complex and has a very medical design
Ethical issues	- No obvious ethical approval
Other	- Looks familiar to industry-led trials and nurses generally do not tend to be involved in planning such trials - No mention of what happens to early termination subjects - Small size set up so CRN would not be in there

## Discussion

We were interested in establishing if involving CRNs early in the trial design could improve trial processes. To test our hypothesis, we gave research nurses existing trial protocols and asked them to make a judgement on the trial status, recruitment and retention statistics and whether or not a CRN was involved in its design. We have shown that from reading a protocol, CRNs are very good judges of trial recruitment, successfully identifying 100% of sites that met their recruitment target, and 100% of the time discerning when trials will not meet their recruitment target. While judgements of trial status and participant retention were not as good, (correct predictions were approximately 50%) we know from prior research work on retention in trials that retention strategies are often poorly specified in trial protocols [16], so it is likely to have been more difficult to make accurate predictions on this. Key to the predictions were the reasons for their judgements. CRNs identified common issues with regard to recruitment such as having an inappropriate recruitment strategy, ill-thought-out timelines and common issues with retention such as high participant burden. These are all issues that can be resolved at the design stage of the protocol. We ask the question hence; would it be beneficial to widen the trial design team to include CRNs?

Five CRNs made judgements on 23 outcome predictions across 18 trials with very good success. These were retrospective predictions. Thus, if these CRNs were involved in the trial design from the outset, and could prospectively contribute, would some of those trials that were terminated or withdrawn have succeeded, participant time not been wasted and potentially new treatments to improve patient outcomes been recommended? We do not know the answer here, but we could put this theory to the test in a prospective study. However, rather than make a recommendation for further studies, we could just take our findings along with the Estimating Site Performance (ESP) [18] findings seriously and broaden our trial design teams. On balance, it surely would do more good than harm.

The trial protocol was frequently mentioned positively as a reason for a successful trial (“simple protocol”) or also as a common reason for a trial withdrawing or terminating (“protocol design seems cumbersome for patients”; “protocol poorly designed”). Similarly, the ESP study [18] reported homogenous  findings, i.e. a burdensome protocol affects recruitment negatively and stated “problems with the trial protocol and/or its implementation” [18] was one of their main reasons why trials fail to recruit. This is an indication of how important the clarity of the trial protocol is for all sites and members of the trial team. Involvement of the CRN in its design could increase its clarity and ease of implementation.

Participant burden was consistently provided as a reason for successful (low burden) or unsuccessful (high burden) trial recruitment, why trials did or did not retain their recruited participants and why the CRN was not involved in the trial. In previous research, the level of perceived burden to a participant in a clinical trial has been divided into five subheadings: “physical, psychological, economic, familial, and social burden” [19]. The higher the level of participant burden, the higher the associated thoughts of withdrawal from a trial. We recently conducted a review of prostate cancer clinical trials and estimated the participant burden as per the above categories. We also found that 50% of trials had a high burden (not published). Why trialists continue to burden patients with copious amounts of data collection is unclear, particularly when the evidence is that much of the data collected during studies is not to support the primary outcome, the most important outcome in the trial. As concluded by the DataCat project [20]: “A small proportion of data collected in the studied trials was related to the primary outcome, while a substantial amount was not related to trial outcomes.” Our study provides further evidence that CRNs are capable of identifying low and high participant burden and subsequent implications for the trial and thus having them as part of the trial design team (in addition to patient and public partners) is important.

The ESP study [18] asked trial managers to predict whether a site would recruit to target and provide reasons for these predictions. Ten trial managers made predictions for 56 site visits recruiting to eight trials. Trial managers correctly predicted the sites that were recruited to target 65% of the time. Our CRNs did slightly better, correctly predicting this 74% of the time. Like our study, the ESP study explored the reasons why the TMs gave the predictions, refining them into 8 red flags. Three of these red flags overlap with our study — “Patient or staff preferences or beliefs”, “Target for recruitment” and “problems with the trial protocol and/or its implementation”. The remaining five flags were specifically site related, and thus not applicable to our study (“previous poor performance”, “Slow/non-standard approval process”, “Lack of engagement of site team”, “Lack of research experience of site staff and staff changes” and “Busy site staff”). The similarities of the relevant themes identified between our two studies should be sufficient reasons to address these red flags when designing clinical trials. CRNs and trial managers play a vital role in this.

## Limitations of the study

Our study is small but this was as large as was feasible for full-time CRNs in a busy clinical research facility. It could be repeated to strengthen the findings but taken alongside the ESP study which had the same findings, we do not believe this is necessary. We asked the CRNs to sign a document stating they did not search the internet while undertaking the study. All CRNs signed the agreement, and we trust their integrity, but we had no way of monitoring their use of the internet as the protocols were read, and the questionnaire answered, outside of work time. Our study is limited by the CRNs reviewing different packs of protocols as some may have been more difficult to assess. It was necessary to do this to avoid contamination bias, as all CRNs work together daily. Additionally, we cannot rule out that the judgements made in a pack of protocols evaluated by the same nurse may not be independent. This is compounded by Pack A being repeated. A larger study using one pack of trial protocols but run across different trial units/facilities would address this limitation.

## Conclusions

Our study and the ESP study should give trialists pause for thought. The two studies used different methodologies but the red flags for trial success that they identified are very similar, reinforcing confidence in the usefulness of the warning signs these flags give. A trial team is exactly that, a team. Engagement of the full team skills from the outset is important if we are to address the issues raised here. Although small, our study and the ESP study are too similar to ignore.

## Supplementary Information

**Additional file 1.** Questionnaire.

**Additional file 2.** STROBE Statement—checklist of items that should be included in reports of observational studies.

## Data Availability

The datasets used and/or analysed during the current study are available from the corresponding author on reasonable request.

## Abbreviations

CRN: Clinical research nurse
PPV: Positive predictive value
NPV: Negative predictive value
