# Supplementary material for: Clinical research nurse predictions of trial failure, recruitment and retention: a case for their early inclusion in trial design
Source: Trials. 2023 Jul 18;24:458. doi: 10.1186/s13063-023-07504-9 (PMC10353190; doi:10.1186/s13063-023-07504-9)
Supplement: Supplementary file 1 — Additional file 1. Questionnaire. [file 13063_2023_7504_MOESM1_ESM.docx]

1. Do you think this study was terminated/withdrawn/completed? Delete the incorrect responses leaving just your response.
   1. Terminated
   2. Withdrawn
   3. Completed
2. Please give a reason for your selection, based on your reading of the protocol.
3. Will this trial successfully recruit?
   1. Yes
   2. No
4. Please give a reason for your answer
5. What proportion of its target do you estimate it will recruit?
   1. 0 - 25%
   2. 25 – 50%
   3. 50 – 75%
   4. 75 – 100%
6. Will it recruit on time/according to the proposed schedule?
   1. Yes
   2. No
7. Please give a reason for your answer
8. Will this trial successfully retain its recruited patients?
   1. Yes
   2. No
9. Please give a reason for your answer
10. Do you think the CRN was involved in the design of this trial?
    1. Yes
    2. No
11. Please give a reason for your answer
12. What other issues/problems do you see for this trial?
